# Brief report on similar mutational changes in neurofibromatosis type 2 gene in minute pulmonary meningothelial-like nodule and meningioma of the central nervous system

**DOI:** 10.18632/oncotarget.26325

**Published:** 2018-11-13

**Authors:** Mitsunori Higuchi, Masayuki Watanabe, Takuya Inoue, Takumi Yamaura, Tomoko Suzuki, Miwako Saito, Katsunao Niitsuma, Kotaro Endo, Ikuro Oshibe, Nobutoshi Soeta, Takuro Saito, Hiroshi Hojo, Mitsuru Munakata, Hiroyuki Suzuki

**Affiliations:** ^1^ Department of Thoracic Surgery, Aizu Medical Center, Fukushima Medical University, Tanisawa, Kawahigashi, Aizuwakamatsu 969-3492, Japan; ^2^ Department of Chest Surgery, Fukushima Medical University School of Medicine, Fukushima 960-1295, Japan; ^3^ Department of Infection and Pulmonary Medicine, Aizu Medical Center, Fukushima Medical University, Tanisawa, Kawahigashi, Aizuwakamatsu 969-3492, Japan; ^4^ Department of Surgery, Aizu Medical Center, Fukushima Medical University, Tanisawa, Kawahigashi, Aizuwakamatsu 969-3492, Japan; ^5^ Department of Pathology, Aizu Medical Center, Fukushima Medical University, Tanisawa, Kawahigashi, Aizuwakamatsu 969-3492, Japan

**Keywords:** minute pulmonary meningothelial-like nodule, meningioma of central nervous system, immunohistochemistry, neurofibromatosis-2 gene, fluorescence in situ hybridization

## Abstract

**Introduction:**

Minute Pulmonary Meningothelial-like Nodules (MPMNs) are usually detected incidentally adjacent to lung cancer tissue. The pathogenesis is unknown. MPMNs reportedly share the status of neurofibromatosis (NF)-2 gene with meningiomas of the central nervous system.

**Results:**

Immunohistochemical staining of two MPMNs revealed they were positive for epithelial membrane antigen (EMA), vimentin, CD56, and progesterone. We identified deletion of the *NF-2* gene in two MPMNs and one CNS meningioma.

**Conclusions:**

MPMN and CNS meningioma may develop via the same mechanism through *NF-2* translocation. Further studies are required to elucidate the genetic similarities between these entities.

**Methods:**

We used fluorescence *in situ* hybridization to explore the status of the *NF-2* gene in MPMNs and compare it with that of CNS meningiomas. We used a commercially available locus-specific probe for the *NF-2* region to analyze whole tissue sections of two MPMNs and two CNS meningiomas by fluorescence *in situ* hybridization.

## INTRODUCTION

Minute pulmonary meningothelial-like nodules (MPMNs) are characteristically asymptomatic, usually being detected incidentally in resected lung specimens. In 1960, Korn and colleagues first described a pulmonary lesion consisting of small nests of cells located in the interstitium and associated with small veins [1]. The authors proposed they have a chemoreceptor cell origin on the basis of their cytologic characteristics, cellular organization, and relationship to vessels, and named them “multiple minute pulmonary tumors resembling chemodectomas”.

In Spain’s series, most of these lesions occurred in patients with pulmonary thromboemboli [2]; ischemia caused by vascular occlusion was therefore considered a possible stimulus for their development from precursor chemoreceptors. However, Kuhn and Askin [3] and Churg and Warnock [4] questioned the chemoreceptor-like nature of these nodules, having demonstrated they have ultrastructural features of meningothelial rather than neuroendocrine cells. Weissferdt and colleagues explored the status of neurofibromatosis (NF)-2 gene in pleuropulmonary meningothelial proliferations and compared it with that of meningiomas of the central nervous system (CNS) [5]. They demonstrated that pleuropulmonary meningothelial lesions have genetic pathways in common with CNS meningiomas and also provided support for the hypothesis that MPMNs and pulmonary meningiomas are related lesions, possibly arising from the same precursor cells. In this study, we used interphase fluorescence *in situ* hybridization (FISH) to assess the *NF-2* status of two MPMNs and two CNS meningiomas.

## RESULTS

### Clinical features

The MPMNs had been found in specimens from one woman and one man, as had the CNS meningiomas. Patients and tumor characteristics and FISH status are summarized in Table 1. One MPMN was an incidental finding during examination of a lung specimen resected for an unrelated adenocarcinoma and the other from a patient with respiratory symptoms, including dry cough and dyspnea, who had wide-spread ground glass opacities. Neither of the study patients with MPMN had clinical or radiological evidence of intracranial or spinal meningiomas or a history of *NF-2-*associated syndromes.

**Table 1:** Clinical and FISH characteristics of two MPMNs and two CNS meningiomas

Case No.	Age	Gender	Diagnosis	Type of treatment	NF-2 gene status (loss of signal)	Prognosis	Recurrence	OS after initial treatment (Months)
1	67	Female	MPMN and lung adenocarcinoma	Left upper lobectomy	Deletion (>50%)	Alive	−	18
2	70	Male	Widespread MPMN only	Conservative	Deletion (>50%)	Alive	−	24
3	39	Male	Pulmonary metastasis of CNS meningioma	Partial resection	Deletion (>50%)	Alive	+	84
4	78	Male	Spinal meningioma (Th3 level)	Extirpation	Normal (15%)	Alive	−	54

### Pathological features

The MPMNs showed interstitial proliferation of cytologically bland oval to blunted spindle cells. Immunohistochemical staining was positive for epithelial membrane antigen (EMA), vimentin, CD56, and progesterone receptor (PgR) in both MPMNs.

### FISH results

**Figure 1:**
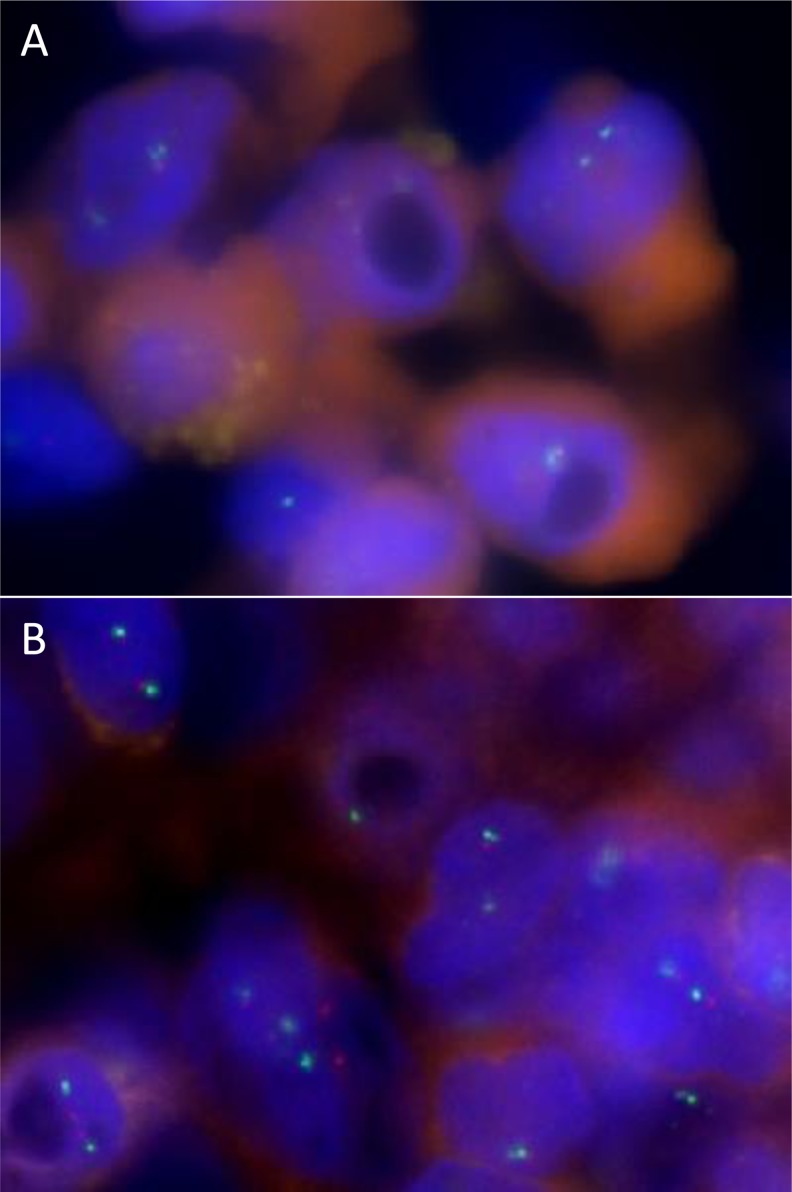
FISH analysis showing deletion of *NF-2* gene in an MPMN (**A**) Loss of one red and one green signal in >50% of tumor cells was interpreted as deletion of 22q. Normal amounts of 22q (two red NF2 and two green CEN22q signals in >50% of nuclei) are also shown (**B**).

FISH analysis was performed on two MPMN and two CNS meningiomas (Table 1). Deletion of *NF-2* was identified in the two MPMNs and one of the two CNS meningiomas (Figure 1). Polysomy of 22q was not identified in this series.

## DISCUSSION

MPMN was first described almost six decades ago; however, the exact origin and pathogenesis of these curious lesions are still unknown. Pulmonary meningothelial-like nodules are considered to be reactive and have been noted to have histologic, immunohistochemical, and ultrastructural features of meningiomas [3, 4, 6–8]. Ionescu *et al.* reported a mutational analysis of MPMNs and intracranial meningiomas and found that isolated MPMNs lack any mutational damage, further supporting a reactive origin [9]. However, the majority of meningiomas showed major molecular events with a high frequency of loss of heterozygosity at 22q. This reported difference in genetic alterations between MPMNs and intracranial meningiomas has been interpreted as supporting the theory that the lesions arise from different cells, have different histogeneses, and progress through different molecular pathways.

Immunohistochemical studies have shown that MPMNs stain positive for EMA, vimentin, and progesterone, and negative for cytokeratin, actin, CD34, S-100 protein, chromogranin, synaptophysin, and neuron-specific enolase [6, 7, 10]. It has also been shown that MPMNs have immunoreactivity for progesterone receptors, suggesting that sex steroids play a role in control of their growth [7]. Furthermore, Mukhipadhyay and colleagues were the first to report positive staining for CD56 [11], which is commonly present in neuroendocrine proliferations and has also been reported in meningiomas [12]. The finding of CD56 staining in MPMN supports meningothelial differentiation.

CNS meningiomas have been found to exhibit a number of genetic aberrations and mutations, including chromosomal abnormalities [13, 14]. Among these, loss of the *NF-2* gene on chromosome 22q has been associated both with meningiomas arising in patients with neurofibromatosis syndrome and in up 60% of sporadic meningiomas [15–17]. Weissferdt *et al.* [5] investigated *NF-2* gene status in six MPMNs, three pleural or pulmonary meningiomas (PPMs), and nine CNS meningiomas. They found two deletions (33.3%) and two polysomes (33.3%) of *NF-2* gene in MPMNs, one deletion (33.3%) and one polysome (33.3%) of *NF-2* gene in PPMs, and four deletions (44.4%) of *NF-2* gene in CNS meningiomas. They have demonstrated that MPMNs and PPMs develop along molecular pathways analogous to those of CNS meningiomas. We have reported here two deletions of *NF-2* gene in two MPMNs and one deletion of *NF-2* gene in two CNS meningiomas. Other support for the hypothesis that MPMNs and pulmonary meningiomas are related lesions and may arise from the same precursor cells has been also reported [5]. Our data are compatible with reported data, although we only studied two MPMNs and this small number is a limitation of this study. In the near future, investigation of *NF-2* gene may lead to molecular targeted therapy for PPMs and CNS meningiomas with MPMN lesions. At this stage, investigation of *NF-2* gene status in MPMNs is interesting, although these are characteristically incidentally detected benign tumors. Accumulation of data about *NF-2* gene status in MPMNs, PPMs and CNS meningiomas is needed to further explore the pathogenesis of these entities.

Niho *et al.* found MPMNs in 10% of patients with lung adenocarcinoma [7]. Mizutani *et al.* also reported that MPMNs are found more often in patients with malignant pulmonary tumors (especially adenocarcinoma) than with other pulmonary disorders [18]. Although most MPMNs are benign lesions with an indolent clinical course, our second patient presented with more extensive atypical MPMN, which was characterized by widespread ground glass attenuation mimicking pulmonary adenocarcinoma *in situ*.

In conclusion, MPMNs may develop via the same mechanism as CNS meningiomas or neurofibromatosis syndrome, through deletion of the *NF-2* gene. More detailed studies are required to elucidate the correlations between these entities and their pathogenesis to enable development of more individualized therapies.

## MATERIALS AND METHODS

### Study specimens

This report describes findings in two MPMNs and two CNS meningiomas found in surgical specimens at Aizu Medical Center, Fukushima Medical University, Aizuwakamatsu and Fukushima Medical University Hospital, Fukushima, Japan. Whole histologic sections from formalin-fixed paraffin-embedded tumor tissue stained with hematoxylin and eosin as well as corresponding unstained slides for FISH were available for all four cases. Clinical information was obtained from the patients’ medical records. This study was approved by the Ethics Committee of Fukushima Medical University (Accession number: 29283). The study was conducted in accordance with the Declaration of Helsinki and Good Clinical Practice guidelines.

### Pathological evaluation

All four specimens were assessed pathologically by examination of hematoxylin and eosin-stained sections. Immunohistochemical staining was also performed on formalin-fixed paraffin-embedded sections of two MPMN specimens using a standard streptavidin-biotin-peroxidase technique with appropriate positive and negative controls. The following antibodies were used: epithelial membrane antigen (EMA) (E29, 1:400; Thermo, Grand Island, NY, USA), CD56 (123C3, 1:400; Invitrogen, Carlsbad, CA, USA), vimentin (V9, 1:1600; Dako, Santa Clara, CA, USA), and progesterone receptor (PgR) (PgR636, undiluted solution; Dako).

### FISH

FISH was performed using a commercially available dual-color DNA probe targeting *NF-2* (22q12.2) colored with Texas Red and CEN22q (22q11.22) colored with FITC (GSP Laboratories, Kobe, Japan), the latter serving as an internal control. Sections of 4 μm thickness were deparaffinized, dehydrated, and air-dried, followed by protease treatment at 37° C for 15 minutes. The slides were then washed in 2× standard saline citrate, dehydrated at room temperature, and allowed to air dry. After marking the hybridization area, the FISH probe was applied to all sections, followed by denaturing of probe and target at 75° C for 5 minutes. Overnight hybridization at 37° C took place in a humidified chamber. Post-hybridization washes in 2× saline sodium citrate (SSC) (5 min, room temperature), 2×SSC/0.3% NP40 (1 to 2 min, 75° C), and 2× SSC (1 min, room temperature) were performed and the slides allowed to air dry. DAPI (4′,6-diamidino-2-phenylindole, 1500 ng/mL; Abnova, Taoyuan, Taiwan) was used as a nuclear counterstain and the sections were examined using a fluorescent microscope with the appropriate filters (Olympus, Melville, NY, USA). One hundred non-overlapping interphase nuclei were scored for the number of fluorescent signals. Hybridizations were considered non-informative if the FISH signals were either lacking or too weak to interpret. Interpretation of deletion required >50% of nuclei containing only one *NF-2* signal, as previously reported [19]. Polysomies (gains) were arbitrarily defined as >5% of nuclei containing three or more signals.
